# Living together—does it help or hinder the pursuit of a healthful diet, physical activity, and weight loss among cancer survivors and their chosen partners?

**DOI:** 10.1007/s00520-024-08907-3

**Published:** 2024-10-03

**Authors:** Harleen Kaur, Gregory Pavela, Dori W. Pekmezi, Laura Q. Rogers, W. Walker Cole, Kelsey B. Parrish, R. Drew Sayer, Holly R. Wyatt, Tracy E. Crane, Hoda Badr, Wendy Demark-Wahnefried

**Affiliations:** 1 Division of Medical Oncology, University of Miami, Miami, FL USA; 2 Department of Health Behavior, University of Alabama at Birmingham, Birmingham, AL USA; 3 Department of Medicine, University of Alabama at Birmingham, Birmingham, AL USA; 4 Department of Family and Community Medicine, University of Alabama at Birmingham, Birmingham, AL USA; 5 Department of Nutrition Sciences, University of Alabama at Birmingham, Birmingham, AL USA; 6 Department of Medicine, Baylor College of Medicine, Houston, TX USA

**Keywords:** Cancer survivors, Cohabitation, Partners, Diet, Weight loss, Physical activity

## Abstract

**Purpose:**

Parental or spousal cohabitating relationships are often targeted in behavioral interventions, but the contribution of cohabitation is poorly understood. This study explored whether cohabitation status moderated the impact of social cognitive theory constructs on adiposity, diet, and exercise in a dyadic, web-based weight loss intervention among cancer survivors and their chosen partners.

**Methods:**

The 24-week weight loss intervention was conducted among 56 dyads, comprised of a cancer survivor and their chosen partner (*n* = 112). Baseline and 6-month data on social cognitive theory constructs (self-efficacy, social support, and perceived barriers), and study outcomes of adiposity (weight and waist circumference), diet (calories and diet quality), and moderate-to-vigorous physical activity (MVPA) were used to perform moderated-mediation analyses among cohabitating (*n* = 25) versus non-cohabitating (*n* = 31) dyads.

**Results:**

The intervention was positively associated with dietary social support (path *a*_1_: 0.36, 95% CI [0.083, 0.659]) and inversely associated with perceived dietary barriers (path *a*_1_: − 10.57, 95% CI [− 19.109, − 3.091]) and perceived exercise barriers (path *a*_1_: − 11.29, 95% CI [− 22.098, − 3.500]) among those who did not cohabitate (effects not observed among cohabitating pairs). The intervention’s mediating effects through perceived barriers on weight (indirect effect (ab): − 2.21, 95% CI [− 4.794, − 0.548]), waist circumference (ab: − 1.13, 95% CI [− 2.452, − 0.171]), caloric intake (ab: − 5.09 (2.86), 95% CI [− 12.602, − 0.709]), and self-reported MVPA (ab: 0.29 (0.18), 95% CI [0.019, 0.754]) also were stronger among non-cohabitating pairs.

**Conclusions:**

These findings suggest that social support partners outside the home substantially enhance intervention impact, though relationship quality and gender dynamics warrant further exploration.

**Trial registration:**

ClinicalTrials.gov Identifier: NCT04132219.

**Supplementary Information:**

The online version contains supplementary material available at 10.1007/s00520-024-08907-3.

## Background

Cancer is the second most prevalent cause of mortality in the USA [1]. However, advancements in early cancer screening and effective therapies have led to increasing numbers of cancer survivors (> 18 million) [2]. The journey of survivorship is accompanied by significant challenges including elevated risks of cancer recurrence, second malignancies, cardiovascular disease, and other comorbidity [3, 4]. These risks are exacerbated by weight gain [5], with reports from 2021 indicating that approximately 32% of cancer survivors have obesity and many more are overweight [6]. Poor dietary and physical activity (PA) behaviors may contribute to the rising obesity rates among cancer survivors [5]. Furthermore, many cancer survivors do not meet the national guidelines, specifically the consumption of five servings of vegetables and fruits (V&Fs) per day or 150 min of moderate-to-vigorous physical activity (MVPA) per week [7]. Hence, there has been an increasing number of web-based lifestyle interventions that use innovative and personalized technologies (e.g., SMS text messaging and websites) to promote beneficial diet and PA behaviors for cancer survivors [8].

Effective lifestyle interventions, whether delivered in-person or by telephone, mailing, and/or web-based platforms, are grounded in behavioral theory to guide diet and PA behavior change with social cognitive theory (SCT) being one of the more robust and heavily used theories [9, 10]. SCT focuses on the reciprocal interplay of constructs such as self-efficacy (confidence in performing behaviors), social support, and the ability to overcome individual and environmental barriers [10]. Previous research has identified the importance of self-efficacy in the initiation, adoption, and maintenance of healthy behavior change [11]. Furthermore, augmenting social support networks (family and friends) and addressing environmental barriers have also been shown to improve diet and PA behaviors [12, 13].

Social relationships are integral to cancer survivors due to the importance of family and friends during cancer diagnosis, treatment, and survivorship [14]. Therefore, behavior change may be more sustainable among survivors when support, feedback, and motivation are shared with others. To leverage these connections, the Daughters, dUdes, mothers, and othErs Together (DUET) trial capitalized on social relationships to target behavioral change [15]. The DUET trial was a dyadic web-based lifestyle intervention implemented among 56 cancer survivor and chosen partner dyads (*n* = 112) to promote diet and PA behaviors and weight loss through an interactive website, fitness trackers, and digital scales [15]. Results indicated that the DUET trial significantly reduced body weight and caloric intake among dyads randomized to the intervention versus the control arms [16]. Given that the DUET intervention was grounded in SCT and incorporated strategies to target self-efficacy, social support, and perceived barriers using online technologies, a mediation analysis was conducted to evaluate these select SCT mechanisms on the intervention’s effects on body weight and related health behaviors [17]. The findings revealed that reductions in perceived dietary barriers mediated the intervention’s effects on weight, waist circumference (WC), and caloric intake, while self-efficacy and social support did not emerge as significant mediators [17].

Cohabitating relationships are a potentially important factor that may simultaneously influence self-efficacy, social support, and barriers to behavior change during lifestyle interventions [18, 19]. Cohabitation likely leads to similar home practices, such as food procurement and preparation and shared daily life activities and responsibilities, which can greatly influence lifestyle behaviors [18]. Cohabitating partners may also bolster social support by sharing similar challenges and resources that facilitate healthier lifestyles [18, 19]. Thus, interventions that include individuals who share similar home environments may bolster social support and self-efficacy and reduce barriers by modeling successful behavior change, overcoming barriers together, and providing a supportive network. Forty-five percent of partners (spouses, children, siblings, friends, etc.) enrolled in the DUET trial cohabitated, while 55% did not. This nearly even distribution presented an opportunity to investigate the potential moderating effect of cohabitation on SCT constructs and within mediating processes.

Given that cohabitating relationships often facilitate shared lifestyle practices and reciprocal support, recent systematic reviews suggest the need to explore the role of cohabitation and the influence of specific support networks’ on healthy lifestyle behaviors [18, 20]. To pioneer these efforts, depicted in Supplementary Information Figure 1, the current study explores whether the effect of the DUET intervention on self-efficacy, social support, and perceived barriers is moderated by cohabitation, and whether the previously shown mediating effects of the DUET intervention on adiposity, diet, and/or MVPA vary by cohabitation status. Given the extant literature, we hypothesized that the intervention’s effects on self-efficacy, social support, and perceived barriers, as well as the mediating indirect effects, would be stronger among cancer survivors and partners who cohabitated versus those who did not.

## Data and methods

### Study design

DUET was a 2-arm, single-blinded, randomized controlled trial in which 56 dyads (comprised of a cancer survivor and their chosen partner, selected from their network of family members, friends, and neighbors) were randomized to a 24-week web-based diet and exercise intervention or to a wait-list control. The study received approval from the University of Alabama at Birmingham (UAB) Institutional Review Board (IRB# 300003882) and was registered with ClinicalTrials.gov (NCT04132219). Written informed consent was obtained from all study participants before enrollment. Details on the methods and primary outcomes of the DUET RCT are published elsewhere [15, 16], including a secondary analysis in which a simple mediation analysis was reported [17]. The current study builds on this mediation analysis.

### Study participants

Cancer survivors with a history of completing treatment for obesity-related early-stage cancers with a 5-year survival rate of ≥ 70% (i.e., female breast, prostate, colorectal, endometrial, localized renal, and loco-regional ovarian) were identified by cancer registries and self-referral networks. Survivors were contacted by telephone and interested participants were screened as follows: (1) age ≥ 18; (2) BMI ≥ 25 kg/m^2^; (3) V&F intake < 2.5 servings/day; (4) MVPA < 150 min/week; (5) access to the Internet and a mobile phone; and (6) could identify a potential partner with whom they interact at least twice weekly to participate in the DUET intervention. Chosen partners were screened using similar eligibility criteria, excluding cancer diagnosis.

### DUET intervention and social cognitive theory mediators

To promote diet and PA behaviors, an interactive website was created that led survivors and their partners through a 24-week series of diet and exercise content that focused on the American Cancer Society and the World Cancer Research Fund-American Institute for Cancer Research guidelines to promote consumption of V&Fs and whole grains, and to decrease consumption of red and processed meats, refined foods, and sugar-sweetened beverages; and performing 150 min/week of MVPA [5, 15]. Messaging focused on SCT constructs, e.g., incremental goal setting with accompanying reinforcement, strategies and role-playing on tackling barriers related to diet and exercise, and self-monitoring to bolster self-efficacy. The weekly sessions also counseled both dyad members on strategies for requesting and offering social support [15, 17]. A text message library encouraged partner support and shepherded dyads back to the website to view new weekly content and log their progress on behavioral goals. The DUET intervention also incorporated Fitbit® Inspire fitness trackers and Aria 2 digital scales (Fitbit, Inc. San Francisco, CA, USA) to assist with self-monitoring. Details on the targeting of SCT constructs are provided in Supplementary Information Table 1.

### Measures

#### Sociodemographic variables

Cancer type and stage were acquired through cancer registries or verification from treating physicians for self-referrals. Baseline sociodemographic variables (e.g., age, sex, race, socio-economic indicators, and dyad relationship status) were collected via self-reported online surveys.

#### Dependent (Y) and mediating variables (M)

Methodological specifications of dependent variables (i.e., weight, WC, caloric intake, diet quality, and MVPA) and mediating variables (i.e., self-efficacy, social support, and perceived barriers) are summarized in Supplementary Information Table 1.

#### Moderating variable (W)

Cohabitation status was determined based on the survivors’ and partners’ mailing addresses (obtained via cancer registries or self-report) and miles between the dyads. Dyad members’ mailing addresses were matched to identify whether the dyads lived together (0 miles between the dyads) or separately (> 0 miles).

#### Statistical analysis

To investigate the moderating effect of cohabitation on self-efficacy, social support, and perceived barriers, moderated mediation analysis was performed on 112 cancer survivors and their partners (56 dyads). As shown in Supplemental Figure 1, the model explores the following: (1) whether the effect of the DUET intervention on self-efficacy, social support, and perceived barriers is moderated by cohabitation (path a); and (2) whether the indirect effects (path a × path b) are conditional on cohabitation. The moderating variable (W), cohabitation status, was coded as a dichotomous variable (0 = do not cohabitate vs 1 = cohabitate). The moderated mediation analysis on baseline and 6-month data was performed using the Mplus structural equation modeling software through a non-parametric resampling approach (version 8, Muthen & Muthen, Los Angeles, CA, USA) to account for independence due to the dyadic nature of this investigation. The moderating effects on path a by cohabitation status are reported as follows: (1) the effect of the DUET intervention (*X*) on self-efficacy, social support, and perceived barriers (*M*) when cohabitation status (*W*) is 0 (path *a*_1_); (2) the effect of cohabitation status on self-efficacy, social support, and perceived barriers when the DUET intervention is 0 (path *a*_2_); and (3) the interaction effect: change in the effect of the DUET intervention on self-efficacy, social support, and perceived barriers as cohabitation status goes from 0 to 1 (path *a*_3_). Conditional effects at the level of the moderator for path *a*_1_ and indirect effects are reported using unstandardized parameter estimates, and standard errors (SE) and statistical significance is determined when the 95% confidence interval (CI) excludes zero. To accommodate the concern for independence and control multilevel data, a clustering syntax was used in each model. Given the potential confounding influence of sex on cohabitation status, sex was used as a covariate in the models. Two sensitivity analyses were conducted to evaluate the robustness of the models given the skewed sex distribution among the cohabitating groups. The first sensitivity analysis involved removing sex as a covariate, which revealed consistency in the unstandardized estimates across the different modeling specifications. Next, sex was examined as a potential effect modifier, which did not show significant moderation effects, ensuring the validity of the findings. Other information on assumptions, covariates, and bootstrapping resamples was detailed previously [17].

## Results

Table 1 represents the study sample by cohabitation status. Across both groups, most survivors were diagnosed with early-stage breast cancer. Furthermore, most identified as non-Hispanic White, resided in urban areas, had a college degree, were employed, and earned an annual income of $50 k or more. Among cohabitating pairs, 92% were spouses with an average age of 61 years. Conversely, among non-cohabitating pairs, 54% were classified as friends, 22% as siblings, and 16% shared a parent–child relationship; most (96.8%) survivors and partners were females, with a mean age of 56 years.

**Table 1:** Characteristics of 112 participants across both study arms, stratified by cohabitation status

	Cohabitated (*n* = 50)	Did not cohabitate (*n* = 62)
Age^a^ [mean (SD); range]	61.0 (11.0) 24–80	56.3 (13.7) 23–81
	*N* (%)	*N* (%)
Study arm		
DUET intervention	24 (42.9%)	32 (57.1%)
Wait-list control	26 (46.4%)	30 (53.6%)
Cancer type		
Breast	16 (32.0%)	33 (53.2%)
Other^b^	11 (22.0%)	3 (4.84%)
N/A^c^	23 (46%)	26 (41.9%)
Cancer stage		
I	11 (22.0%)	12 (19.3%)
II	4 (8.0%)	2 (3.2%)
III	1 (2.0%)	0 (0%)
Unknown	34 (68.0%)	48 (77.4%)
Relationship among dyads		
Spouse	23 (92%)	0 (0%)
Child	1 (4%)	5 (16.1%)
Sibling	0 (0%)	7 (22.6%)
Friend	0 (0%)	17 (54.8%)
Other^d^	1 (4%)	2 (6.5%)
Sex		
Female	26 (52.0%)	60 (96.8%)
Male	24 (48.0%)	2 (3.2%)
Race		
Non-Hispanic White	32 (64.0%)	38 (61.3%)
Non-Hispanic Black	18 (36.0%)	23 (37.1%)
Other^e^	0 (0%)	1 (1.6%)
Residence		
Urban	43 (86.0%)	60 (96.8%)
Rural	7 (14.0%)	2 (3.2%)
Education		
High school diploma or lower	5 (10.0%)	11 (17.7%)
Some college	12 (24.0%)	22 (35.5%)
College graduate or more	31 (62.0%)	29 (46.8%)
Refused/unknown	2 (4.0%)	0 (0%)
Employment		
Employed	31 (62.0%)	31 (50.0%)
Retired	17 (34.0%)	19 (30.7%)
Other^f^	2 (4.0%)	12 (19.4%)
Income		
< $50 k/year	2 (4.0%)	16 (25.8%)
≥ $50 k/year	27 (54.0%)	21 (33.9%)
Refused/unknown	21 (42.0%)	25 (40.3%)

^a^Age quantified in years; ^b^Other cancer types: prostate, colorectal, gynecological, and renal; ^c^*N/A*, not applicable category as it represents the partner group who were cancer free; ^d^Other relationship types: parent, son-in-law, and neighbor; ^e^Hispanics and other racial groups; ^f^Other employment: student and disabled

In all models, the intervention effect was significantly associated with improvements in dietary social support (Table 2, significant path *a*_1_) among those who did not cohabitate, significant conditional path *a*_1_ in Table 3. Moreover, in the diet quality model, cohabitation moderated the effect of the intervention on dietary social support (interaction effect (*a*_3_) =  − 0.45 (0.23), 95% CI [− 0.940, − 0.003]), such that participants who did not cohabitate reported significantly higher social support compared to those who lived together. However, cohabitation did not play a moderating role in the indirect effects. No significant moderating effects were found on exercise social support.

**Table 2:** Moderated mediation analysis: exploring whether the effect of the DUET intervention on social support is moderated by cohabitation status

Antecedent	Consequent							
		Dietary social support (*M*)				Weight (Y)		
		Coeff.^a^	SE^b^	95% CI^c^		Coeff	SE	95% CI
DUET vs WLC^d^ (*X*)	*a*_1_	0.36	0.15	0.083, 0.659*	*c*′^e^	− 3.14	1.90	− 6.632, 0.509
Dietary social support (*M*)		-	-	-	*b*^f^	− 0.76	1.51	− 3.314, 2.825
Cohabitation (*W*)	*a*_2_	0.53	0.21	0.153, 0.939		-	-	-
Interaction (*X* × *W*)	*a*_3_	− 0.43	0.25	− 0.922, 0.005		-	-	-
Constant	*i*_M_^g^	1.77	0.77	0.310, 3.159	*i*_Y_^h^	15.15	10.24	− 6.637, 32.860
		*R*^2^ = 0.357, *p* = < 0.001				*R*^2^ = 0.945, *p* = < 0.001		
Antecedent	Consequent							
		Dietary social support (*M*)				Waist circumference (Y)		
		Coeff.^a^	SE^b^	95% CI^c^		Coeff	SE	95% CI
DUET vs WLC (*X*)	*a*_1_	0.37	0.15	0.100, 0.677*	*c*′	− 1.10	1.08	− 3.119, 1.052
Dietary social support (*M*)		-	-	-	*b*	− 0.50	0.91	− 2.231, 1.296
Cohabitation (*W*)	*a*_2_	0.56	0.21	0.108, 0.958		-	-	-
Interaction (*X* × *W*)	*a*_3_	− 0.44	0.24	− 0.929, 0.001		-	-	-
Constant	*i*_M_	1.37	0.81	− 0.023, 3.225	*i*_Y_	9.51	9.27	− 8.760, 26.491
		*R*^2^ = 0.362, *p* = < 0.001				*R*^2^ = 0.850, *p* = < 0.001		
Antecedent	Consequent							
		Dietary social support (*M*)				Calories (Y)		
		Coeff.^a^	SE^b^	95% CI^c^		Coeff	SE	95% CI
DUET vs WLC (*X*)	*a*_1_	0.34	0.15	0.092, 0.677*	*c*′	− 12.51	7.70	− 28.254, 0.975
Dietary social support (*M*)		-	-	-	*b*	5.98	4.37	− 1.732, 16.634
Cohabitation (*W*)	*a*_2_	0.54	0.21	0.109, 0.949		-	-	-
Interaction (*X* × *W*)	*a*_3_	− 0.42	0.24	− 0.932, 0.016		-	-	-
Constant	*i*_M_	2.02	0.52	1.117, 2.956	*i*_Y_	72.21	28.74	13.993, 125.782
		*R*^2^ = 0.354, *p* = < 0.001				*R*^2^ = 0.475, *p* = < 0.001		
Antecedent	Consequent							
		Dietary social support (*M*)				Diet quality (Y)		
		Coeff.^a^	SE^b^	95% CI^c^		Coeff	SE	95% CI
DUET vs WLC (*X*)	*a*_1_	0.36	0.14	0.099, 0.643*	*c*′	3.64	2.79	− 1.620, 5.991
Dietary social support (*M*)		-	-	-	*b*	2.74	1.87	− 1.413, 9.448
Cohabitation (*W*)	*a*_2_	0.60	0.21	0.228, 1.055		-	-	-
Interaction (*X* × *W*)	*a*_3_	− 0.45	0.23	− 0.940, − 0.003*		-	-	-
Constant	*i*_M_	1.46	0.43	0.726, 2.397	*i*_Y_	26.36	12.02	2.943, 48.732
		*R*^2^ = 0.378, *p* = < 0.001				*R*^2^ = 0.132, *p* = 0.179		
Antecedent	Consequent							
		Exercise social support (*M*)				Self-report MVPA^i^ (Y)		
		Coeff.^a^	SE^b^	95% CI^c^		Coeff	SE	95% CI
DUET vs WLC (*X*)	*a*_1_	0.30	0.18	− 0.056, 0.645*	*c*′	0.70	0.42	− 0.047, 1.547
Exercise social support (*M*)		-	-	-	*b*	0.69	0.27	0.193, 1.238
Cohabitation (*W*)	*a*_2_	0.26	0.20	− 0.203, 0.609		-	-	-
Interaction (*X* × *W*)	*a*_3_	− 0.25	0.28	− 0.832, 0.273		-	-	-
Constant	*i*_M_	1.29	0.55	0.207, 2.341	*i*_Y_	− 0.93	1.33	− 3.958, 1.203
		*R*^2^ = 0.277, *p* = 0.001				*R*^2^ = 0.284, *p* = 0.002		
Antecedent	Consequent							
		Exercise social support (*M*)				Accelerometer MVPA (Y)		
		Coeff.^a^	SE^b^	95% CI^c^		Coeff	SE	95% CI
DUET vs WLC (*X*)	*a*_1_	0.38	0.23	− 0.037, 0.888*	*c*′	0.27	0.42	− 0.602, 1.026
Exercise social support (*M*)		-	-	-	*b*	0.55	0.22	0.153, 1.003
Cohabitation (*W*)	*a*_2_	0.19	0.24	− 0.394, 0.605		-	-	-
Interaction (*X* × *W*)	*a*_3_	− 0.39	0.36	− 1.019, 0.357		-	-	-
Constant	*i*_M_	1.59	0.77	0.158, 3.133	*i*_Y_	1.50	1.41	− 1.412, 4.797
		*R*^2^ = 0.288, *p* = 0.003				*R*^2^ = 0.356, *p* = < 0.001		

^*a*^*Coeff*., unstandardized parameter estimates; ^*b*^*SE*, standard errors; ^*c*^*CI*, confidence interval, statistically significant associations when 95% CI excludes zero (*); ^*d*^*WLC*, wait-list control arm; ^*e*^*c*′, direct effect: the effect of the independent variable on the dependent variable (controlling for the mediator); ^*f*^*b*, the effect of the mediator on the dependent variable conditional on the moderator while controlling for the independent variable; ^g^*i*_M_, intercept; ^*h*^*i*_*Y*_, intercept; ^*i*^*MVPA*, moderate-to-vigorous physical activity

**Table 3:** Conditional effects of the DUET intervention on social support and conditional indirect effects at dichotomized values of the moderator

	Path a^a^		Path b^b^	Indirect effect^c^		
Paths	Cohabitated (Coeff.^d^, SE^e^, 95%CI^f^)	Did not Cohabitate (Coeff., SE, 95%CI)	b (Coeff., SE, 95%CI)	Cohabitated (Coeff., SE, 95%CI)	Did not cohabitate (Coeff., SE, 95%CI)	Index of moderated mediation (Coeff., SE, 95%CI)
DUET vs WLC^g^ $\to$ dietary social support $\to$ weight	− 0.07 (0.19) [− 0.455, 0.297]	0.36 (0.15)* [0.083, 0.659]	− 0.76 (1.51) [− 3.314, 2.825]	0.06 (0.33) [− 0.431, 0.972]	− 0.27 (0.61) [− 1.577, 0.915]	0.33 (0.76) [− 0.936, 2.185]
DUET vs WLC $\to$ dietary social support $\to$ waist circumference	− 0.07 (0.20) [− 0.473, 0.296]	0.37 (0.15)* [0.100, 0.677]	− 0.50 (0.91) [− 2.231, 1.296]	0.03 (0.22) [− 0.197, 0.684]	− 0.18 (0.40) [− 1.132, 0.541]	0.22 (0.48) [− 0.568, 1.571]
DUET vs WLC $\to$ dietary social support $\to$ calories	− 0.08 (0.19) [− 0.497, 0.257]	0.34 (0.15)* [0.092, 0.677]	5.98 (4.37) [− 1.732, 16.634]	− 0.48 (1.31) [− 3.775, 1.227]	2.05 (1.70) [− 0.101, 6.856]	− 2.53 (2.17) [− 9.565, 0.244]
DUET vs WLC $\to$ dietary social support $\to$ diet quality	− 0.09 (0.18) [− 0.454, 0.264]	0.36 (0.14)* [0.099, 0.643]	2.74 (1.87) [− 1.413, 9.448]	− 0.26 (0.64) [− 2.136, 0.676]	0.98 (0.80) [− 0.132, 3.179]	− 1.23 (1.15) [− 4.774, 0.138]
DUET vs WLC $\to$ exercise social support $\to$ MVPA^h^	0.05 (0.24) [− 0.390, 0.503]	0.30 (0.18)* [− 0.056, 0.645]	0.69 (0.27) [0.193, 1.238]	0.04 (0.20) [− 0.292, 0.478]	0.21 (0.18) [− 0.016, 0.680]	− 0.17 (0.23) [− 0.762, 0.146]
DUET vs WLC $\to$ exercise social support $\to$ accelerometer MVPA	− 0.01 (0.28) [− 0.462, 0.631]	0.38 (0.23)* [− 0.037, 0.888]	0.55 (0.22) [0.153, 1.003]	− 0.01 (0.16) [− 0.306, 0.379]	0.21 (0.15) [− 0.001, 0.607]	− 0.22 (0.23) [− 0.780, 0.149]

^a^Path a = the effect of the independent variable on the mediator by cohabitation status, ${\theta }_{X\to M}={a}_{1}+{a}_{3}W$; ^b^Path b = the effect of the mediator on the dependent variable conditional on the moderator while controlling for the independent variable; ^c^Indirect effects obtained by taking the product of path a and path b by cohabitating status, ${b\theta }_{X\to M}=b({a}_{1}+{a}_{3}W)$; ^*d*^*Coeff*., unstandardized parameter estimates; ^*e*^*SE*, standard errors; ^*f*^*CI*, confidence interval, statistically significant associations when 95% CI excludes zero (*); ^*g*^*WLC*, wait-list control arm; ^*h*^*MVPA*, self-reported moderate-to-vigorous physical activity

Table 4 shows the moderated effect of cohabitation on perceived barriers. In all models, the intervention effect was significantly associated with reductions in dietary and exercise barriers (significant path *a*_1_) among those who did not cohabitate, significant conditional path *a*_1_ in Table 5. However, cohabitation did not moderate the effect of the DUET intervention on perceived barriers (non-significant path *a*_3_). The moderated mediation analysis, depicted in Table 5, revealed that for weight, WC, and caloric intake models, conditional mediating effects for dietary barriers were significantly stronger among those who did not live together (− 2.21 (1.09), 95% CI [− 4.794, − 0.548]), (− 1.13 (0.58), 95% CI [− 2.452, − 0.171]), and (− 5.09 (2.86), 95% CI [− 12.602, − 0.709]), respectively. However, the index of moderation revealed that these conditional mediating effects were not significantly different between cohabitating groups. A significantly stronger conditional mediating effect for exercise barriers (0.29 (0.18), 95% CI [0.019, 0.754]) was also found among those who did not cohabitate in the self-reported MVPA model. The index of moderation (− 0.31 (0.22), 95% CI [− 0.879, − 0.008]) revealed that this conditional mediating effect was significantly different among the cohabitating groups, though these results were not corroborated in the accelerometer-measured MVPA model.

**Table 4:** Moderated mediation analysis: exploring whether the effect of the DUET intervention on perceived barriers is moderated by cohabitation status

Antecedent	Consequent							
		Dietary barriers (*M*)				Weight (Y)		
		Coeff.^a^	SE^b^	95% CI^c^		Coeff	SE	95% CI
DUET vs WLC^d^ (*X*)	*a*_1_	− 10.57	3.82	− 19.109, − 3.091*	*c*′^e^	− 1.53	2.06	− 5.657, 2.242
Dietary barriers (*M*)		-	-	-	*b*^f^	0.21	0.06	0.084, 0.314*
Cohabitation (*W*)	*a*_2_	− 1.89	3.91	− 9.586, 6.568		-	-	-
Interaction (*X* × *W*)	*a*_3_	6.78	5.58	− 4.394, 18.141		-	-	-
Constant	*i*_M_^g^	34.52	14.88	5.436, 64.721	*i*_Y_^h^	6.41	8.28	-10.807, 21.181
		*R*^2^ = 0.288, *p* = 0.001				*R*^2^ = 0.951, *p* = < 0.001		
Antecedent	Consequent							
		Dietary barriers (*M*)				Waist circumference (Y)		
		Coeff.^a^	SE^b^	95% CI^c^		Coeff	SE	95% CI
DUET vs WLC (*X*)	*a*_1_	− 10.36	3.78	− 18.787, − 3.246*	*c*′	− 0.46	1.16	− 2.673, 1.821
Dietary barriers (*M*)		-	-	-	*b*	0.11	0.04	0.012, 0.158*
Cohabitation (*W*)	*a*_2_	− 3.52	3.93	− 10.594, 5.297		-	-	-
Interaction (*X* × *W*)	*a*_3_	6.56	5.47	− 4.068, 17.310		-	-	-
Constant	*i*_M_	45.66	16.41	14.778, 79.464	*i*_Y_	6.22	7.22	− 8.530, 19.250
		*R*^2^ = 0.289, *p* = < 0.001				*R*^2^ = 0.862, *p* = < 0.001		
Antecedent	Consequent							
		Dietary barriers (*M*)				Calories (Y)		
		Coeff.^a^	SE^b^	95% CI^c^		Coeff	SE	95% CI
DUET vs WLC (*X*)	*a*_1_	− 8.34	3.82	− 16.182, − 0.905*	*c*′	− 7.68	7.62	− 23.373, 5.472
Dietary barriers (*M*)		-	-	-	*b*	0.61	0.17	0.280, 0.943*
Cohabitation (*W*)	*a*_2_	− 2.47	3.85	− 10.332, 4.631		-	-	-
Interaction (*X* × *W*)	*a*_3_	5.96	5.46	− 4.846, 17.510		-	-	-
Constant	*i*_M_	4.15	8.39	− 12.665, 20.372	*i*_Y_	69.06	22.70	17.671, 112.124
		*R*^2^ = 0.260, *p* = 0.002				*R*^2^ = 0.497, *p* = < 0.001		
Antecedent	Consequent							
		Dietary barriers (*M*)				Diet quality (Y)		
		Coeff.^a^	SE^b^	95% CI^c^		Coeff	SE	95% CI
DUET vs WLC (*X*)	*a*_1_	− 8.87	3.76	− 16.223, − 1.328*	*c*′	3.39	3.03	− 2.136, 9.317
Dietary barriers (*M*)		-	-	-	*b*	− 0.10	0.09	− 0.260, 0.061
Cohabitation (*W*)	*a*_2_	− 1.61	3.93	− 9.500, 5.791		-	-	-
Interaction (*X* × *W*)	*a*_3_	5.56	5.40	− 5.345, 16.887		-	-	-
Constant	*i*_M_	10.22	8.56	− 5.712, 30.036	*i*_Y_	38.46	10.54	15.989, 57.577
		*R*^2^ = 0.247, *p* = 0.004				*R*^2^ = 0.111, *p* = 0.223		
Antecedent	Consequent							
		Exercise barriers (*M*)				Self-report MVPA^i^ (Y)		
		Coeff.^a^	SE^b^	95% CI^c^		Coeff	SE	95% CI
DUET vs WLC (*X*)	*a*_1_	− 11.29	4.76	− 22.098, − 3.500*	*c*′	0.70	0.43	− 0.030, 1.555
Exercise barriers (*M*)		-	-	-	*b*	− 0.03	0.01	− 0.056, − 0.003*
Cohabitation (*W*)	*a*_2_	− 4.20	6.28	− 17.835, 7.209		-	-	-
Interaction (*X* × *W*)	*a*_3_	12.07	6.94	− 0.238, 26.013		-	-	-
Constant	*i*_M_	15.16	9.39	− 4.079, 33.381	i_Y_	2.56	1.34	− 0.152, 4.982
		*R*^2^ = 0.297, *p* = 0.004				*R*^2^ = 0.312, *p* = 0.001		
Antecedent	Consequent							
		Exercise barriers (*M*)				Accelerometer MVPA (Y)		
		Coeff.^a^	SE^b^	95% CI^c^		Coeff	SE	95% CI
DUET vs WLC (*X*)	*a*_1_	− 11.48	5.58	− 24.350, − 1.891*	c′	0.32	0.44	− 0.611, 1.083
Exercise barriers (*M*)		-	-	-	b	− 0.01	0.01	− 0.029, 0.014
Cohabitation (*W*)	*a*_2_	− 4.77	7.10	− 18.583, 8.565		-	-	-
Interaction (*X* × *W*)	*a*_3_	12.21	8.16	− 2.655, 29.991		-	-	-
Constant	*i*_M_	22.08	13.21	− 2.855, 48.274	i_Y_	2.31	1.35	-0.699, 4.871
		*R*^2^ = 0.295, *p* = 0.007				*R*^2^ = 0.329, *p* = 0.002		

^*a*^*Coeff*., unstandardized parameter estimates; ^*b*^*SE*, standard errors; ^*c*^*CI*, confidence interval, statistically significant associations when 95% CI excludes zero (*); ^*d*^*WLC*, wait-list control arm; ^*e*^*c*′, direct effect: the effect of the independent variable on the dependent variable (controlling for the mediator); ^*f*^*b*, the effect of the mediator on the dependent variable conditional on the moderator while controlling for the independent variable; ^g^i_M_, intercept; ^*h*^*i*_*Y*_, intercept; ^*i*^*MVPA*, moderate-to-vigorous physical activity

**Table 5:** Conditional effects of the DUET intervention on perceived barriers and conditional indirect effects at dichotomized values of the moderator

	Path a^a^		Path b^b^	Indirect Effect^c^		
Paths	Cohabitated (Coeff.^d^, SE^e^, 95%CI^f^)	Did not cohabitate (Coeff., SE, 95%CI)	b (Coeff., SE, 95%CI)	Cohabitated (Coeff., SE, 95%CI)	Did not cohabitate (Coeff., SE, 95%CI)	Index of moderated mediation (Coeff., SE, 95%CI)
DUET vs WLC^g^ $\to$ dietary barriers $\to$ weight	− 3.79 (4.01) [− 11.855, 3.800]	− 10.57 (3.82)* [− 19.109, -3.091]	0.21 (0.06) [0.084, 0.314]	− 0.79 (0.90) [− 3.620, 0.628]	− 2.21 (1.09)* [− 4.794, − 0.548]	1.42 (1.33) [− 0.706, 4.485]
DUET vs WLC $\to$ dietary barriers $\to$ waist circumference	− 3.80 (3.81) [− 12.302, 3.451]	− 10.36 (3.78)* [− 18.787, − 3.246]	0.11 (0.04) [0.012, 0.158]	− 0.42 (0.45) [− 1.572, 0.341]	− 1.13 (0.58)* [− 2.452, − 0.171]	0.72 (0.71) [− 0.460, 2.280]
DUET vs WLC $\to$ dietary barriers $\to$ calories	− 2.38 (3.46) [− 9.155, 4.367]	− 8.34 (3.82)* [− 16.182, − 0.905]	0.61 (0.17) [0.280, 0.943]	− 1.45 (2.09) [− 5.178, 3.005]	− 5.09 (2.86)* [− 12.602, − 0.709]	3.64 (3.79) [− 2.081, 13.643]
DUET vs WLC $\to$ dietary barriers $\to$ diet quality	− 3.32 (3.60) [− 10.812, 3.649]	− 8.87 (3.76)* [− 16.223, − 1.328]	− 0.10 (0.09) [− 0.260, 0.061]	0.32 (0.48) [− 0.285, 1.663]	0.85 (0.86) [− 0.187, 3.303]	− 0.53 (0.90) [− 4.004, 0.241]
DUET vs WLC $\to$ exercise barriers $\to$ self-report MVPA^h^	0.78 (5.14) [− 9.009, 12.666]	− 11.29 (4.76)* [− 22.098, − 3.500]	− 0.03 (0.01) [− 0.056, − 0.003]	− 0.02 (0.14) [− 0.390, 0.229]	0.29 (0.18)* [0.019, 0.754]	− 0.31 (0.22)* [− 0.879, − 0.008]
DUET vs WLC $\to$ exercise barriers $\to$ accelerometer MVPA	0.73 (5.82) [− 10.468, 12.249]	− 11.48 (5.58)* [− 24.350, − 1.891]	− 0.01 (0.01) [− 0.029, 0.014]	− 0.00 (0.08) [− 0.207, 0.085]	0.05 (0.14) [− 0.169, 0.438]	− 0.06 (0.16) [− 0.734, 0.146]

^a^Path a = the effect of the independent variable on the mediator by cohabitation status, ${\theta }_{X\to M}={a}_{1}+{a}_{3}W$; ^b^Path b = the effect of the mediator on the dependent variable conditional on the moderator while controlling for the independent variable; ^c^Indirect effects obtained by taking the product of path a and path b by cohabitating status, ${b\theta }_{X\to M}=b({a}_{1}+{a}_{3}W)$; ^*d*^*Coeff*., unstandardized parameter estimates; ^*e*^*SE*, standard errors; ^*f*^*CI*, confidence interval, statistically significant associations when 95% CI excludes zero (*); ^*g*^*WLC*, wait-list control arm; ^*h*^*MVPA*, self-reported moderate-to-vigorous physical activity

Table 6 shows the moderated effect of cohabitation on self-efficacy. Cohabitation did not moderate the intervention effect on self-efficacy (non-significant path *a*_1_ effects in each model), and cohabitation did not moderate the indirect effects (non-significant path b in each model).

**Table 6:** Moderated mediation analysis: exploring whether the effect of the DUET intervention on self-efficacy is moderated by cohabitation status

Antecedent	Consequent							
		Dietary self-efficacy (*M*)				Weight (Y)		
		Coeff.^a^	SE^b^	95% CI^c^		Coeff	SE	95% CI
DUET vs WLC^d^ (*X*)	*a*_1_	0.02	0.05	− 0.075, 0.126	*c*′^e^	− 2.18	1.98	− 6.178, 1.464
Dietary self-efficacy (*M*)		-	-	-	*b*^f^	− 13.44	7.93	− 27.417, 3.969
Cohabitation (*W*)	*a*_2_	− 0.04	0.05	− 0.139, 0.045		-	-	-
Interaction (*X* × *W*)	*a*_3_	0.08	0.07	− 0.030, 0.205		-	-	-
Constant	*i*_M_^g^	0.14	0.19	− 0.209, 0.506	*i*_Y_^h^	10.65	8.24	− 6.051, 27.144
		*R*^2^ = 0.355, *p* = < 0.001				*R*^2^ = 0.949, *p* = < 0.001		
Antecedent	Consequent							
		Dietary self-efficacy (*M*)				Waist circumference (Y)		
		Coeff.^a^	SE^b^	95% CI^c^		Coeff	SE	95% CI
DUET vs WLC (*X*)	*a*_1_	0.02	0.05	− 0.074, 0.122	*c*′	− 0.91	1.18	− 3.503, 1.198
Dietary self-efficacy (*M*)		-	-	-	*b*	− 4.65	3.66	− 11.697, 1.995
Cohabitation (*W*)	*a*_2_	− 0.04	0.05	− 0.141, 0.052		-	-	-
Interaction (*X* × *W*)	*a*_3_	0.08	0.06	− 0.029, 0.203		-	-	-
Constant	*i*_M_	0.14	0.23	− 0.289, 0.595	*i*_Y_	11.66	8.10	− 5.079, 26.345
		*R*^2^ = 0.351, *p* = < 0.001				*R*^2^ = 0.849, *p* = < 0.001		
Antecedent	Consequent							
		Dietary self-efficacy (*M*)				Calories (Y)		
		Coeff.^a^	SE^b^	95% CI^c^		Coeff	SE	95% CI
DUET vs WLC (*X*)	*a*_1_	0.001	0.05	− 0.090, 0.099	*c*′	− 11.57	8.13	− 27.774, 2.437
Dietary self-efficacy (*M*)		-	-	-	*b*	− 36.18	20.52	− 74.778, 2.653
Cohabitation (*W*)	*a*_2_	− 0.03	0.05	− 0.137, 0.058		-	-	-
Interaction (*X* × *W*)	*a*_3_	0.08	0.06	− 0.032, 0.200		-	-	-
Constant	*i*_M_	0.44	0.14	0.158, 0.691	*i*_Y_	110.71	38.23	34.350, 179.191
		*R*^2^ = 0.371, *p* = < 0.001				*R*^2^ = 0.484, *p* = < 0.001		
Antecedent	Consequent							
		Dietary self-efficacy (*M*)				Diet quality (Y)		
		Coeff.^a^	SE^b^	95% CI^c^		Coeff	SE	95% CI
DUET vs WLC (*X*)	*a*_1_	0.01	0.05	− 0.076, 0.104	*c*′	3.40	2.92	− 2.023, 9.030
Dietary self-efficacy (*M*)		-	-	-	*b*	10.99	8.09	− 6.721, 27.459
Cohabitation (*W*)	*a*_2_	− 0.06	0.05	− 0.170, 0.032		-	-	-
Interaction (*X* × *W*)	*a*_3_	0.09	0.06	− 0.027, 0.214		-	-	-
Constant	*i*_M_	0.37	0.14	0.103, 0.623	*i*_Y_	33.26	10.17	11.725, 52.956
		*R*^2^ = 0.356, *p* = < 0.001				*R*^2^ = 0.117, *p* = 0.179		
Antecedent	Consequent							
		Exercise self-efficacy (*M*)				Self-report MVPA^i^ (Y)		
		Coeff.^a^	SE^b^	95% CI^c^		Coeff	SE	95% CI
DUET vs WLC (*X*)	*a*_1_	0.09	0.06	− 0.025, 0.221	*c*′	0.83	0.39	0.111, 1.674
Exercise self-efficacy (*M*)		-	-	-	*b*	1.32	1.00	− 0.582, 3.301
Cohabitation (*W*)	*a*_2_	− 0.09	0.07	− 0.238, 0.058		-	-	-
Interaction (*X* × *W*)	*a*_3_	− 0.11	0.09	− 0.291, 0.051		-	-	-
Constant	i_M_	0.27	0.14	− 0.008, 0.538	*i*_Y_	− 0.46	1.29	− 3.241, 1.870
		*R*^2^ = 0.271, *p* = 0.004				*R*^2^ = 0.336, *p* = < 0.001		
Antecedent	Consequent							
		Exercise self-efficacy (*M*)				Accelerometer MVPA (Y)		
		Coeff.^a^	SE^b^	95% CI^c^		Coeff	SE	95% CI
DUET vs WLC (*X*)	*a*_1_	0.11	0.08	− 0.020, 0.274	*c*′	0.32	0.40	− 0.595, 0.988
Exercise self-efficacy (*M*)		-	-	-	*b*	1.10	0.77	− 0.426, 2.491
Cohabitation (*W*)	*a*_2_	− 0.08	0.09	− 0.251, 0.112			-	-
Interaction (*X* × *W*)	*a*_3_	− 0.13	0.11	− 0.338, 0.067		-	-	-
Constant	*i*_M_	0.29	0.22	− 0.135, 0.689	*i*_Y_	1.13	1.40	− 1.491, 4.143
		*R*^2^ = 0.301, *p* = 0.005				*R*^2^ = 0.356, *p* = < 0.001		

^*a*^*Coeff*., unstandardized parameter estimates; ^*b*^*SE*, standard errors; ^*c*^*CI*, confidence interval, statistically significant associations when 95% CI excludes zero; ^*d*^*WLC*, wait-list control arm; ^*e*^*c*′, direct effect: the effect of the independent variable on the dependent variable (controlling for the mediator); ^*f*^*b*, the effect of the mediator on the dependent variable conditional on the moderator while controlling for the independent variable; ^*g*^*i*_*M*_, intercept; ^*h*^*i*_*Y*_, intercept; ^*i*^*MVPA*, moderate-to-vigorous physical activity

## Discussion

Cohabitation has been shown to influence lifestyle behaviors [18, 19], yet there is limited understanding of the moderating effects of cohabitation within dyadic lifestyle interventions. Our investigation is unique as it is the first dyadic trial which explores the moderated-mediated role of cohabitation. We hypothesized that individuals who cohabitated and shared a similar home environment would show greater improvements in self-efficacy, social support, and reductions in diet and exercise barriers and that these mediating effects would be stronger among cohabitating pairs. However, our data did not support this hypothesis. Instead, individuals who did not cohabitate showed significant improvements in dietary social support and reported fewer dietary and exercise barriers. Furthermore, mediating effects through perceived barriers were stronger among cancer survivors and partners who did not cohabitate. No moderated mediation effect was found for self-efficacy.

To our knowledge, no other research studies have used a similar approach to examine the role of cohabitation on social support. However, previous reports offer insights that validate our findings. Notably, dietary social support was significantly higher among cancer survivors and partners who did not cohabitate. A potential explanation for this finding could be that non-cohabitating pairs may have provided more focused support by engaging in more purposeful communication about dietary goals and behaviors. Lewis [21] and Umberson [22] propose that the quality of communication impacts the quantity of social support among partners. The distance between dyad members may have therefore initiated intentional support efforts, resulting in clearer communication. Additionally, given that most cohabitating dyads were in spousal relationships, social support provided by the spouse may be perceived as social control [23–25]. Health-related social control refers to various interpersonal strategies (pressure, guilt, negotiation, etc.) to influence another’s health behaviors [26]. While social control has been shown to promote healthy behaviors [22], its effect is highly dependent on relationship quality (not measured in this study) [21, 27, 28]. Therefore, within the context of this study, non-cohabitating dyads may have shared friendship relationships with less conflict and more supportive conversations regarding achieving dietary goals.

Another important consideration is dependency on social support networks by gender as research shows that men are more inclined to receive emotional support from women; however, women depend on other social networks (friends, family members, and neighbors) to seek health-related support [19, 29, 30]. In the current study with 96% of females in the non-cohabitating group, female partners may have been better equipped to understand the emotional support required by their counterparts to facilitate dietary change.

Similarly, perceived dietary and exercise barriers were significantly lower and the mediating effects observed for perceived barriers were stronger among non-cohabitating dyads. While there is limited empirical data to support these findings, the principles of temporal self-regulation theory (TST) and self-determination theory (SDT) can help elucidate our results. The TST framework is based on intentions, prepotency, and self-regulatory constructs to explain behavior change [31]. However, an important consideration of this theory is the role of supportive and distractive environments in regulating behavior [31]. Booker and colleagues tested the TST framework which indicated that participants in environments perceived as supportive versus distracting were better able to sustain healthy lifestyle behaviors [32]. In our study, dyads with separate living spaces may have more control over their environment and temptations and therefore show lower dietary and exercise barriers. In other words, those who do not cohabitate may have more autonomy to build a supportive environment for themselves that meets their individual lifestyle needs (e.g., food preferences, types of foods purchased, flexibility in managing workout schedules, and types of exercise performed) as compared to cohabitating partners who may have to account for their partner’s routines, needs, and preferences. Moreover, autonomy, a key consideration of the SDT, has been well-studied in promoting behavior change among the general population, as well as among cancer survivors [33–36]. Based on the SDT, autonomy provides choice and freedom for individuals to regulate their behaviors to achieve a desired outcome [34–37]. Conversely, previous reports show that overweight patients are also dependent on external control to undermine autonomy and improve accountability [38], though autonomous dependence on supportive social networks is also acknowledged [39]. Moreover, those who do not cohabitate may feel more comfortable disclosing weight-loss-related barriers with friends and acquaintances than with immediate family members, perceiving their friends as more empathetic and non-judgmental [40]. Additionally, those who do not live together may have a more objective vantagepoint and an ability to provide clarity, insights, and perspectives that may enhance problem-solving.

Unlike social support and perceived barriers, our findings did not demonstrate that cohabitation moderated self-efficacy in resisting high-calorie foods or performing exercise. Thus. confidence in one’s ability to make healthier diet and exercise choices may be more dependent on personal intentions and motivation [41, 42]. Therefore, reliance on a partner may not be sufficient to target self-efficacy directly. Renner and colleagues corroborate the claim that intentions play a moderating effect on self-efficacy and vary by risk and motives [43]. Cancer survivors, who are at higher risk for cancer recurrence and second primaries, may consequently have distinct intentions and motivations to change their behaviors that differ from their partners, regardless of where their partner resides. Moreover, overcoming ingrained habits and navigating personal environmental influences may be more difficult and complex for cancer survivors due to the impact of cancer diagnosis and treatment, further impacting self-efficacy [29, 44]. Additionally, psychological barriers such as low self-esteem, negative body image, emotional eating, or depression also can alter self-efficacy [29]. While these factors were not measured in the present study, tailored strategies that consider psychological well-being and comprehensive support systems to target self-efficacy are warranted.

### Study strengths and limitations

This study has several strengths, as it is the first, to our knowledge, to investigate the moderated-mediated role of cohabitation in a dyadic-based weight loss intervention. One of the primary strengths of the study is the application of rigorous and thorough structural equation modeling to account for interdependence. By design, very few weight loss trials have been able to perform such analysis, amplifying the importance of our findings for future dyadic and social network interventions. Hence, this study contributes to the limited literature on the role of social networks and the home environment in guiding behavior change and helps inform future dyadic interventions on whether to enroll supportive partners living in the same home environment to maximize impact. The DUET study also included various social support relationships such as spouses, children, siblings, parents, and friends, contributing to the understanding of cohabitation dynamics. While this study provides valuable contributions to the field of social-behavioral research, there were limitations. The study’s shortcoming lies in its secondary analysis, limiting the scope of the investigation. For instance, specific variables (e.g., relationship quality and family dynamics) that may help explain the moderated-mediation effect of cohabitation on SCT constructs were not collected. While sensitivity tests supported the validity of the findings, sparse data on sex may not fully account for its impact in the models. Additionally, the investigation was not powered to perform a comprehensive dyadic analysis (i.e., actor-partner mediator model) to understand how cancer survivors and partners influence each other’s self-efficacy, social support, and perceived barriers. Notably, our findings are specific to a cohort of cancer survivors and their partners who are also of higher socioeconomic status, limiting the generalizability of these findings to all cancer survivors, especially those who are disadvantaged and may rely more on their cohabitating partners to navigate healthful lifestyle change.

### Conclusions

More people are living alone, and increasing trends in loneliness and obesity among cancer survivors and other populations require creative solutions [6, 45]. Home-based interventions that incorporate the support of social networks to promote healthful diet and PA behaviors using online technologies may help lower the burden of obesity. Our findings suggest that social support networks outside the household may offer more effective support and problem-solving strategies than partners who live together. In clinical practice, healthcare providers should tailor diet and PA recommendations based on the unique needs of the cancer survivors’ support networks. Moreover, future research should assess the quality of the relationship between cancer survivors and their partners to improve the impact of dyadic-based interventions.

## Supplementary Information

Below is the link to the electronic supplementary material.

Supplementary file1 (DOCX 30 KB)

## Data Availability

The datasets, materials, and/or codes analyzed in the present study can be obtained from the corresponding author upon reasonable request.

## References

[1] Siegel RL, Miller KD, Fuchs HE, Jemal A (2022) Cancer statistics, 2022. CA Cancer J Clin 72(1):7–33. 10.3322/caac.21708 35020204 10.3322/caac.21708

[2] Miller KD, Nogueira L, Devasia T, Mariotto AB, Yabroff KR, Jemal A, Kramer J, Siegel RL (2022) Cancer treatment and survivorship statistics, 2022. CA Cancer J Clin 72(5):409–436. 10.3322/caac.21731 35736631 10.3322/caac.21731

[3] Sung H, Hyun N, Leach CR, Yabroff KR, Jemal A (2020) Association of first primary cancer with risk of subsequent primary cancer among survivors of adult-onset cancers in the United States. JAMA 324(24):2521–2535. 10.1001/jama.2020.23130 33351041 10.1001/jama.2020.23130 PMC7756242

[4] Warner DF, Schiltz NK, Stange KC, Given CW, Owusu C, Berger NA, Koroukian SM (2017) Complex multimorbidity and health outcomes in older adult cancer survivors. Fam Med Community Health 5(2):129–138. 10.15212/FMCH.2017.0127 30956969 10.15212/FMCH.2017.0127 PMC6450549

[5] Rock CL, Thomson CA, Sullivan KR, Howe CL, Kushi LH, Caan BJ, Neuhouser ML, Bandera EV, Wang Y, Robien K, Basen-Engquist KM, Brown JC, Courneya KS, Crane TE, Garcia DO, Grant BL, Hamilton KK, Hartman SJ, Kenfield SA et al (2022) American Cancer Society nutrition and physical activity guideline for cancer survivors. CA Cancer J Clin 72(3):230–262. 10.3322/caac.21719 35294043 10.3322/caac.21719

[6] National Cancer Institute (2024) Cancer trends progress report: cancer survivors and weight. https://progressreport.cancer.gov/after/weight. Accessed 1 March 2024

[7] Byrd DA, Agurs-Collins T, Berrigan D, Lee R, Thompson FE (2017) Racial and ethnic differences in dietary intake, physical activity, and body mass index (BMI) among cancer survivors: 2005 and 2010 National Health Interview Surveys (NHIS). J Racial Ethn Health Disparities 4(6):1138–1146. 10.1007/s40615-016-0319-8 28078657 10.1007/s40615-016-0319-8

[8] Williams V, Brown N, Becks A, Pekmezi D, Demark-Wahnefried W (2020) Narrative review of web-based healthy lifestyle interventions for cancer survivors. Ann Rev Res 5(4):555670. 10.19080/arr.2020.05.555670 33294850 10.19080/arr.2020.05.555670 PMC7720895

[9] Bluethmann SM, Bartholomew LK, Murphy CC, Vernon SW (2017) Use of theory in behavior change interventions. Health Educ Behav 44(2):245–253. 10.1177/1090198116647712 27226430 10.1177/1090198116647712 PMC5503486

[10] Bandura A (1998) Health promotion from the perspective of social cognitive theory. Psychol Health 13(4):623–649. 10.1080/08870449808407422

[11] Stacey FG, James EL, Chapman K, Courneya KS, Lubans DR (2015) A systematic review and meta-analysis of social cognitive theory-based physical activity and/or nutrition behavior change interventions for cancer survivors. J Cancer Surviv 9(2):305–338. 10.1007/s11764-014-0413-z 25432633 10.1007/s11764-014-0413-z PMC4441740

[12] Latkin CA, Knowlton AR (2015) Social network assessments and interventions for health behavior change: a critical review. Behav Med 41(3):90–97. 10.1080/08964289.2015.1034645 26332926 10.1080/08964289.2015.1034645 PMC4786366

[13] Rogers LQ, Markwell S, Hopkins-Price P, Vicari S, Courneya KS, Hoelzer K, Verhulst S (2011) Reduced barriers mediated physical activity maintenance among breast cancer survivors. J Sport Exerc Psychol 33(2):235–254. 10.1123/jsep.33.2.235 21558582 10.1123/jsep.33.2.235 PMC3145412

[14] Usta YY (2012) Importance of social support in cancer patients. Asian Pac J Cancer Prev 13(8):3569–3572. 10.7314/apjcp.2012.13.8.3569 23098436 10.7314/apjcp.2012.13.8.3569

[15] Pekmezi DW, Crane TE, Oster RA, Rogers LQ, Hoenemeyer T, Farrell D, Cole WW, Wolin K, Badr H, Demark-Wahnefried W (2021) Rationale and methods for a randomized controlled trial of a dyadic, web-based, weight loss intervention among cancer survivors and partners: the DUET Study. Nutrients 13(10):3472. 10.3390/nu13103472 34684474 10.3390/nu13103472 PMC8539255

[16] Demark-Wahnefried W, Oster RA, Crane TE, Rogers LQ, Cole WW, Kaur H, Farrell D, Parrish KB, Badr HJ, Wolin KY, Pekmezi DW (2023) Results of DUET: a web-based weight loss randomized controlled feasibility trial among cancer survivors and their chosen partners. Cancers 15(5):1577. 10.3390/cancers15051577 36900368 10.3390/cancers15051577 PMC10000640

[17] Kaur H, Pavela G, Pekmezi DW, Rogers LQ, Cole WW, Parrish KB, Sayer RD, Wyatt HR, Demark-Wahnefried W (2023) Dietary barriers appear to influence the effects of a dyadic web-based lifestyle intervention on caloric intake and adiposity: a mediation analysis of the DUET trial. Nutrients 15(23):4918. 10.3390/nu15234918 38068776 10.3390/nu15234918 PMC10708365

[18] Werneck AO, Winpenny EM, Foubister C, Guagliano JM, Monnickendam AG, van Sluijs EMF, Corder K (2020) Cohabitation and marriage during the transition between adolescence and emerging adulthood: a systematic review of changes in weight-related outcomes, diet and physical activity. Prev Med Rep 20:101261. 10.1016/j.pmedr.2020.101261 33344148 10.1016/j.pmedr.2020.101261 PMC7736988

[19] Burke TJ, Segrin C (2014) Examining diet- and exercise-related communication in romantic relationships: associations with health behaviors. Health Commun 29(9):877–887. 10.1080/10410236.2013.811625 24295060 10.1080/10410236.2013.811625

[20] Ellis KR, Raji D, Olaniran M, Alick C, Nichols D, Allicock M (2022) A systematic scoping review of post-treatment lifestyle interventions for adult cancer survivors and family members. J Cancer Surviv 16(2):233–256. 10.1007/s11764-021-01013-x 33713302 10.1007/s11764-021-01013-x PMC8564800

[21] Lewis MA, McBride CM, Pollak KI, Puleo E, Butterfield RM, Emmons KM (2006) Understanding health behavior change among couples: an interdependence and communal coping approach. Soc Sci Med 62(6):1369–1380. 10.1016/j.socscimed.2005.08.006 16146666 10.1016/j.socscimed.2005.08.006

[22] Umberson D, Crosnoe R, Reczek C (2010) Social relationships and health behavior across life course. Annu Rev Sociol 36:139–157. 10.1146/annurev-soc-070308-120011 21921974 10.1146/annurev-soc-070308-120011 PMC3171805

[23] Franks MM, Stephens MAP, Rook KS, Franklin BA, Keteyian SJ, Artinian NT (2006) Spouses’ provision of health-related support and control to patients participating in cardiac rehabilitation. J Fam Psychol 20(2):311–318. 10.1037/0893-3200.20.2.311 16756407 10.1037/0893-3200.20.2.311

[24] Burke TJ, Segrin C (2016) Weight-related social control and relationship quality: accuracy and bias effects. J Soc Pers Relat 33(8):999–1017. 10.1177/0265407515615692

[25] Scholz U, Stadler G, Berli C, Lüscher J, Knoll N (2021) How do people experience and respond to social control from their partner? Three daily diary studies. Front Psychol 11:613546. 10.3389/fpsyg.2020.613546 33519637 10.3389/fpsyg.2020.613546 PMC7838347

[26] Lewis MA, Butterfield RM, Darbes LA, Johnston-Brooks C (2004) The conceptualization and assessment of health-related social control. J Soc Pers Relat 21(5):669–687. 10.1177/0265407504045893

[27] Lewis MA, Butterfield RM (2005) Antecedents and reactions to health-related social control. Pers Soc Psychol Bull 31(3):416–427. 10.1177/0146167204271600 15657456 10.1177/0146167204271600

[28] Young VJ, Burke TJ, Curran MA (2019) Interpersonal effects of health-related social control: positive and negative influence, partner health transformations, and relationship quality. J Soc Pers Relat 36(11–12):3986–4004. 10.1177/0265407519846565

[29] Terranova CO, Lawler SP, Spathonis K, Eakin EG, Reeves MM (2017) Breast cancer survivors’ experience of making weight, dietary and physical activity changes during participation in a weight loss intervention. Support Care Cancer 25(5):1455–1463. 10.1007/s00520-016-3542-2 27988868 10.1007/s00520-016-3542-2

[30] Gurung RA, Taylor SE, Seeman TE (2003) Accounting for changes in social support among married older adults: insights from the MacArthur Studies of Successful Aging. Psychol Aging 18(3):487–496. 10.1037/0882-7974.18.3.487 14518810 10.1037/0882-7974.18.3.487

[31] Hall PA, Fong GT (2007) Temporal self-regulation theory: a model for individual health behavior. Health Psychol Rev 1(1):6–52. 10.1080/17437190701492437

[32] Booker L, Mullan B (2013) Using the temporal self-regulation theory to examine the influence of environmental cues on maintaining a healthy lifestyle. Br J Health Psychol 18(4):745–762. 10.1111/bjhp.12015 23279265 10.1111/bjhp.12015

[33] Powers TA, Koestner R, Denes A, Cornelius T, Gorin AA (2022) Autonomy support in a couples weight loss trial: helping yourself while helping others. Fam Syst Health 40(1):70–78. 10.1037/fsh0000663 34855419 10.1037/fsh0000663 PMC9380832

[34] Wilson PM, Blanchard CM, Nehl E, Baker F (2006) Predicting physical activity and outcome expectations in cancer survivors: an application of Self-Determination Theory. Psychooncology 15(7):567–578. 10.1002/pon.990 16304621 10.1002/pon.990

[35] Ntoumanis N, Ng JYY, Prestwich A, Quested E, Hancox JE, Thøgersen-Ntoumani C, Deci EL, Ryan RM, Lonsdale C, Williams GC (2021) A meta-analysis of self-determination theory-informed intervention studies in the health domain: effects on motivation, health behavior, physical, and psychological health. Health Psychol Rev 15(2):214–244. 10.1080/17437199.2020.1718529 31983293 10.1080/17437199.2020.1718529

[36] Gillison FB, Rouse P, Standage M, Sebire SJ, Ryan RM (2019) A meta-analysis of techniques to promote motivation for health behaviour change from a self-determination theory perspective. Health Psychol Rev 13(1):110–130. 10.1080/17437199.2018.1534071 30295176 10.1080/17437199.2018.1534071

[37] Flannery M (2017) Self-Determination theory: intrinsic motivation and behavioral change. Oncol Nurs Forum 44(2):155–156. 10.1188/17.Onf.155-156 28222078 10.1188/17.ONF.155-156

[38] Hardcastle S, Hagger MS (2011) “You Can’t Do It on Your Own”: experiences of a motivational interviewing intervention on physical activity and dietary behaviour. Psychol Sport Exerc 12(3):314–323. 10.1016/j.psychsport.2011.01.001

[39] Gettens KM, Carbonneau N, Koestner R, Powers TA, Gorin AA (2018) The role of partner autonomy support in motivation, well-being, and weight loss among women with higher baseline BMI. Fam Syst Health 36(3):347–356. 10.1037/fsh0000362 29999341 10.1037/fsh0000362 PMC6131030

[40] Wang ML, Pbert L, Lemon SC (2014) Influence of family, friend and coworker social support and social undermining on weight gain prevention among adults. Obesity (Silver Spring) 22(9):1973–1980. 10.1002/oby.20814 24942930 10.1002/oby.20814 PMC4435839

[41] Conner M, Norman P (2022) Understanding the intention-behavior gap: the role of intention strength. Front Psychol 13:923464. 10.3389/fpsyg.2022.923464 35992469 10.3389/fpsyg.2022.923464 PMC9386038

[42] Hardcastle SJ, Hancox J, Hattar A, Maxwell-Smith C, Thøgersen-Ntoumani C, Hagger MS (2015) Motivating the unmotivated: how can health behavior be changed in those unwilling to change? Front Psychol 6:835. 10.3389/fpsyg.2015.00835 26136716 10.3389/fpsyg.2015.00835 PMC4468355

[43] Renner B, Schwarzer R (2005) The motivation to eat a healthy diet: how intenders and nonintenders differ in terms of risk perception, outcome expectancies, self-efficacy, and nutrition behavior. Pol Psychol Bull 36(1):7–15

[44] Balneaves LG, Van Patten C, Truant TL, Kelly MT, Neil SE, Campbell KL (2014) Breast cancer survivors’ perspectives on a weight loss and physical activity lifestyle intervention. Support Care Cancer 22(8):2057–2065. 10.1007/s00520-014-2185-4 24633590 10.1007/s00520-014-2185-4

[45] Snell KDM (2017) The rise of living alone and loneliness in history. Soc Hist 42(1):2–28. 10.1080/03071022.2017.1256093

