# Supplementary material for: Living together—does it help or hinder the pursuit of a healthful diet, physical activity, and weight loss among cancer survivors and their chosen partners?
Source: Support Care Cancer. 2024 Oct 3;32(10):700. doi: 10.1007/s00520-024-08907-3 (PMC11450071; doi:10.1007/s00520-024-08907-3)
Supplement: Supplementary file 1 — Supplementary file1 (DOCX 30 KB) [file 520_2024_8907_MOESM1_ESM.docx]

Self-Efficacy

Social Support

Perceived Barriers (M)

Intervention effect (DUET vs. wait-list control) (X)

Adiposity, Diet, and MVPA (Y)

Path a

Path b

Path c’

Cohabitation (W)

Indirect effect = path a*path b

Supplementary Information Figure 1: Moderated mediation model: Evaluating whether cohabitation status moderates the effect of the DUET web-based lifestyle intervention on self-efficacy, social support, and perceived barriers and whether the indirect effects are conditional on cohabitation status.

Supplementary Information Table 1: Brief description of the DUET dependent and mediating variables. Detailed methods are outlined previously [17].

| **Dependent Variables** | **How it was Assessed** | **How it was Measured** |
| --- | --- | --- |
| Adiposity | Adiposity was assessed remotely at baseline and 6 months by measuring body weight and waist circumference (WC) via Zoom® (San Jose, CA, USA). Weight was assessed using a scale, and waist circumference was assessed using a set of ribbons. | Weight and WC measurements were taken twice and averaged for analysis. |
| Diet | Dietary intake was assessed at baseline and 6 months through two-day dietary recalls. | Diet quality and average caloric intake were measured using the Healthy Eating Index 2015 (HEI-2015). |
| MVPA | Moderate-to-vigorous physical activity (MVPA) was assessed at baseline and 6 months using both objective and subjective methods: ActiGraph accelerometers and the Godin Leisure Time Exercise Questionnaire (GLTEQ), respectively. | Objective MVPA was processed and measured using ActiLife software. Subjective MVPA was measured by summing the moderate and vigorous physical activity items from the GLTEQ. |
| **Mediating Variables** | **How it was Targeted** | **How it was Assessed and Measured** |
| Self-Efficacy | Self-efficacy was targeted through the delivery of weekly interactive e-learning sessions through the DUET website. The sessions covered topics on healthy eating, exercise, and goal setting, while also providing personalized progress reports and tools such as menu plans, tracking forms, and tip sheets to help participants manage diet and physical activity. Interactive video sessions promoted self-efficacy through role modeling, while self-monitoring technologies, including the Inspire Fitbit® and Aria 2 scale, boosted confidence by allowing participants to track and visualize their progress over time. | Self-efficacy for diet was assessed using a 20-item instrument across five subscales: negative emotions, availability, social pressure, physical discomfort, and positive activities.  Self-efficacy for exercise was assessed using the 8-item instrument under various conditions (e.g., lack of discipline, bad weather, lack of time). Average scores were used to measure self-efficacy. |
| Social Support | Social support was enhanced through a buddy-system approach, where both survivors and their partners were guided on how to request and provide support to each other for achieving healthy eating and physical activity goals. The interactive e-learning sessions covered key types of social support (emotional, informational, appraisal, and instrumental), and these were reinforced through assignments built upon teamwork and SMS text messages. | Social support for diet and exercise was assessed using a validated 4-item instrument, which measures participation, involvement, and encouragement. Average scores were used to measure social support. |
| Perceived Barriers | Perceived barriers were targeted by offering evidence-based information through interactive e-learning sessions on dietary and physical activity recommendations tailored for cancer survivors. These sessions provide foundational knowledge on reducing diet and exercise barriers. They cover topics such as goal setting to limit sugar and processed meats, increasing fruit and vegetable intake, managing portion sizes, safe weight loss, and achieving recommended exercise levels. Additionally, the sessions addressed common barriers such as fatigue, stress, time constraints, and access to healthy foods. Features like BMI calculators, meal plans, and exercise logs further supported users by providing practical tools to overcome these challenges. | Barriers diet and exercise were assessed using a 10-item and a 21-item questionnaire, respectively. The percentage of responses was summed to measure perceived barriers. |
